# Population-level analyses identify host and environmental variables influencing the vaginal microbiome

**DOI:** 10.1038/s41392-025-02152-8

**Published:** 2025-02-19

**Authors:** Lang Qin, Tianyong Sun, Xiao Li, Shigang Zhao, Zheng Liu, Changlong Zhang, Congcong Jin, Yanqi Xu, Xuan Gao, Yongzhi Cao, Jiaojiao Wang, Ting Han, Lei Yan, Jialun Song, Fangfang Zhang, Feifei Liu, Yousheng Zhang, Yuzhen Huang, Yuping Song, Yanjun Liu, Jing Zhang, Xiuqing Zhang, Zhina Yao, Honglei Chen, Zhenzhen Zhang, Shengrui Zhao, Yuhan Feng, Ya-nan Zhang, Qian Yu, Fang Cao, Lijuan Zhao, Lei Xie, Ling Geng, Qiang Feng, Han Zhao, Zi-Jiang Chen

**Affiliations:** 1 State Key Laboratory of Reproductive Medicine and Offspring Health, Center for Reproductive Medicine, Institute of Women, Children and Reproductive Health, Shandong University, Jinan, Shandong 250012 China; 2 Key Laboratory of Reproductive Endocrinology (Shandong University), Ministry of Education, Jinan, Shandong 250012 China; 3 Shandong Key Laboratory of Reproductive Research and Birth Defect Prevention, Jinan, Shandong 250012 China; 4 Shandong Technology Innovation Center for Reproductive Health, Jinan, Shandong 250012 China; 5 National Research Center for Assisted Reproductive Technology and Reproductive Genetics, Shandong University, Jinan, Shandong 250012 China; 6 Department of Human Microbiome, School and Hospital of Stomatology, Cheeloo College of Medicine, Shandong University, Jinan, 250012 Shandong China; 7 Shandong Provincial Key Laboratory of Oral Tissue Regeneration, Jinan, 250012 Shandong China; 8 Shandong Engineering Laboratory for Dental Materials and Oral Tissue Regeneration, Jinan, 250012 Shandong China; 9 Reproductive Medicine Center of the First Affiliated Hospital of Wenzhou Medical University, Wenzhou, 325000 Zhejiang China; 10 Linyi People’s Hospital, Linyi, 276000 Shandong China; 11 Department of Obstetrics and Gynecology, Shandong Provincial Hospital Affiliated to Shandong First Medical University, Jinan, 250021 Shandong China; 12 State Key Laboratory of Microbial Technology, Shandong University, Qingdao, China; 13 Shanghai Key Laboratory for Assisted Reproduction and Reproductive Genetics, Shanghai, 200135 China; 14 Department of Reproductive Medicine, Ren Ji Hospital, Shanghai Jiao Tong University School of Medicine, Shanghai, 200135 China

**Keywords:** Reproductive disorders, Genome informatics

## Abstract

The vaginal microbiome is critical for the reproductive health of women, yet the differential impacts exerted by the host and by ambient environmental variables on the vaginal microbiome remain largely unknown. Here, we conducted a comprehensive cross-sectional study of the relationships between the vaginal microbiome and 81 matched host and environmental variables across 6755 Chinese women. By 16S rRNA sequencing, we identified four core vaginal microbiota with a prevalence of over 90% and a total median abundance of 98.8%. Twenty-four variables, including physiology, lifestyle behaviors, gynecologic history, social and environmental information, were found associated with the microbiome composition, of which bacterial vaginosis (BV) showed the largest effect size. Age was among the strongest explanatory variables and the vaginal microbiome dynamically succeeded with increasing age, especially with a composition turning point at the age of 45. Our mediation analyses indicated that the effects of age on the microbiome could be mediated by variables such as parity number and lifestyles. We further classified the vaginal microbiomes of the population into 13 “Vagitypes”. Women with *Lactobacillus iners*- and *Lactobacillus jensenii*-dominated Vagitypes had significantly higher live birth rate than those with Vagitype dominated by *Fannyhessea vaginae* (53.40%, 59.09% vs 21.43%; OR [95% CI]: 3.62 [1.12–14.87], 5.39 [1.27–27.36]; *P* = 0.031, *P* = 0.021). This study provides a comprehensive overview of the associations between identified variables and the vaginal microbiome, representing an important step toward understanding of environment-microbe-host interactions.

## Introduction

The vaginal microbiome plays a crucial role in maintaining the health of reproductive-age women. It is typically dominated by lactic acid-producing *Lactobacillus* species,^1,2^ which help maintain an acidic environment that inhibits the growth of pathogenic bacteria. However, when the abundance of *Lactobacillus* decreases, a condition known as bacterial vaginosis (BV)^3^ can develop. This imbalance not only disrupts the natural defense of the vagina but also increases the risk of various health issues. Greater microbial diversity and dysbiosis in the vagina has been associated with increased susceptibility to sexually transmitted infections,^4,5^ pelvic inflammatory disease,^6^ and additional deleterious impacts on reproductive health.^7,8^

Despite the significant role of the vaginal microbiome in women’s health, a large portion of its variance remains unexplained. Previous research has identified various endogenous and exogenous factors that can influence the composition of the vaginal microbiome, such as sexual activity, hygiene practices, and antibiotic use.^2,9–11^ However, the heterogeneity of study pipelines and the considerable inter-individual variability in microbiome composition pose challenges to the reproducibility of findings across different studies.

In addition, much effort has been devoted to characterizing the compositional patterns of the complex vaginal microbial communities.^2,12–14^ One of the earliest studies was carried out among North American women and defined five vaginal community state types (CSTs).^2^ Four of them are dominated by *Lactobacillus* spp. (*L. crispatus*, CST I; *L. gasseri*, CST II; *L. iners*, CST III; *L. jensenii*, CST V) and one consisting of diverse strict and facultative anaerobes (CST IV). Recently, the vaginal microbial communities were further dissected into thirteen subtypes.^13^ However, the vaginal microbial stratification had been largely investigated in non-Asian populations, and limited association between human phenotype and different microbial community types were provided.

**Fig. 1:**
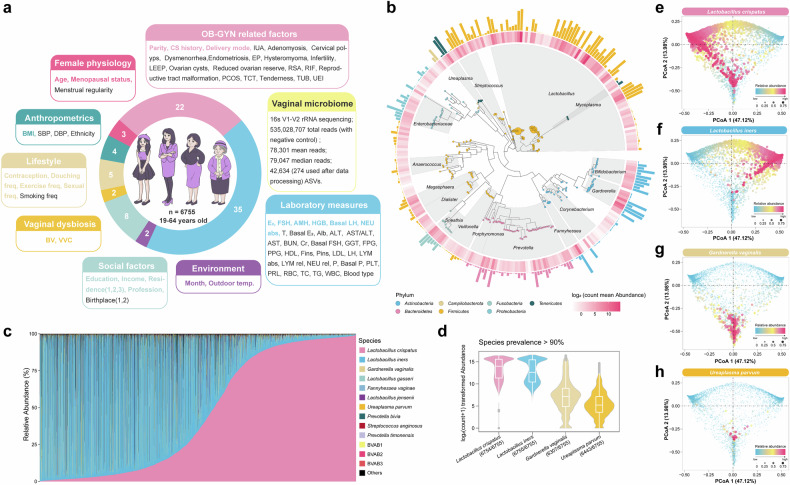
Overview of the study and defining core vaginal microbiome in the VaMHP cohort. **a** 6755 individuals aged 19–64 years were recruited. Vaginal swabs with 81 time-matched variables from eight categories were collected. Wedge sizes in the ring chart reflect the proportion of collected variables in each category. **b** The phylogenetic tree constructed by the 274 representative sequences used in the study. The transparency of colors in the inner heatmap and the heights of bars in the outer ring correspond to the relative abundance of ASVs. The colors of bars in the outer ring represent the Phylum classification of microbes. The abundance of ASVs was transformed using a log_2_(count+1) function. **c** Relative abundances of top 13 taxa across all the individuals’ samples. Each sample is represented by one stacked bar. The bars for all samples were presented in the ascending order of relative abundance of *Lactobacillus crispatus*. “Others” refers to the remaining taxa. **d** The relative abundances of four core species that were observed in over 90% of the sampled participants. The abundances of the species were transformed by log_2_(count+1). **e**–**h** Principal coordinate analysis (PCoA) plots based on Bray–Curtis distance. Each point represents a sample and is colored according to the relative abundance of specific bacterial taxa

Given the highly personal and individualized nature of the vaginal microbiome, large-scale analysis of a standardized cohort with integrated extensive clinical and environmental data is of fundamental value in identifying novel and robust associations. Such studies can reduce the effects of confounding biological factors and provide a more comprehensive understanding of the vaginal microbiome’s role in health and disease.^15,16^ To gain a complete overview of the taxonomic composition of the human vaginal microbiome and to better understand the ecology of the vaginal microbiome and its relationship with the host, we conducted a cross-sectional study using 16S-ribosomal RNA (rRNA)-sequencing on 6755 women from the cohort of the Vaginal Microbiome Health Project (VaMHP) (supplementary Fig. 1a). At least 50,000 reads of each sample were generated and the median sequencing read depth was 79,047. A total of 274 amplicon sequence variants (ASVs) were included in the final analysis (Fig. 1a, supplementary Fig. 1a). The vaginal microbiome was investigated through standardized clinical sample collection and processing procedures. Eight categories of 81 time-matched variables including demographic information, lifestyle behaviors, socioeconomic status, environmental data, gynecological factors, clinical blood profile, female physiology, and vaginal dysbiosis (Fig. 1a, supplementary Table 1, see “Methods”) were analyzed. This comprehensive approach allowed us to explore the complex interplay between the vaginal microbiome and various factors that may influence its composition and stability. Furthermore, we followed up a subset of 845 women to assess the relationship between the vaginal microbiome and the pregnancy outcomes. By investigating these relationships, we aimed to shed light on the potential implications of the vaginal microbiome for reproductive health and identify factors that could be targeted for interventions.

## Results

### The core vaginal microbiome in the VaMHP cohort

Participants were aged 19–64 years (mean age of 33.9 ± 6.3 years old) and had a body mass index (BMI) of 24.0 ± 3.7 kg/m^2^. 16S-rRNA sequencing revealed microbial taxonomic clades ranging from 48 genera (Fig. 1b). Consistent with the previously reported vaginal microbiome,^2^ our analysis of the Chinese cohort indicated that most of the reads came from *Lactobacillus* (Fig. 1b, c). An analysis seeking those microbial taxa that are ubiquitous across the cohort (i.e., the core microbiome^17^) revealed that four microbial species (Fig. 1d, supplementary Table 2) were identified in more than 90% of the examined individuals. Two of them were from the genus *Lactobacillus*, namely *L. crispatus*, and *L. iners*, constituting as high as 90.6% of the total reads. Notably, *L. iners* was detected in all of the examined individuals. *L. crispatus*, the most beneficial probiotic for vaginal health,^18,19^ was the second most prevalent species and was detected in all but one of the examined individuals. The remaining core species comprised the previously reported major BV pathogen *Gardnerella vaginalis*^20^ and the common urogenital tract commensal species *Ureaplasma parvum*.^21^ Principal coordinates analysis (PCoA) revealed that remarkable inter-individual microbial community dissimilarities were driven by the core species (Fig. 1e–h). Collectively, these four bacteria accounted for 98.8% (ranging widely from 1.4% to 100%) of the median abundance and comprised the species-level core vaginal microbiome in our cohort.

### Variables associated with microbial diversity and composition

**Fig. 2:**
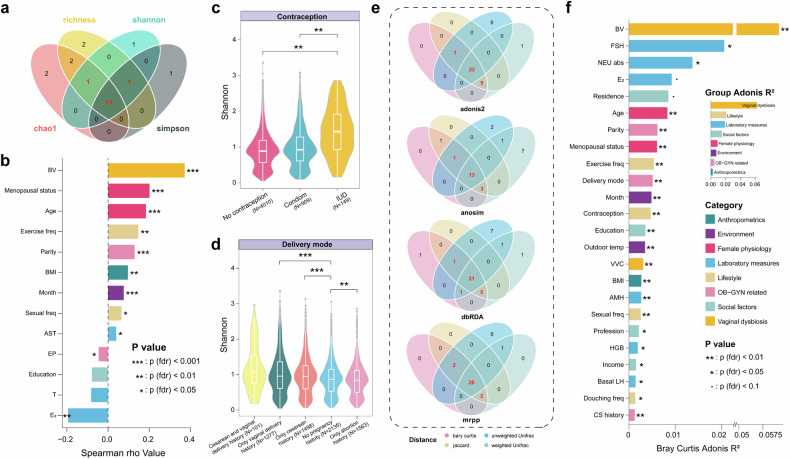
Significant associations between microbiome diversity and host variables. **a** Venn diagram showing the continuous variables identified as significantly associated with four alpha-diversity measures. **b** Variables significantly associated with at least three alpha-diversity measures (FDR < 0.05), represented by Shannon index. Asterisks indicate FDR < 0.05 in the linear regression models. Each factor is colored by its category. BV Bacterial vaginosis, BMI Body mass index, AST Aspartate aminotransferase, EP Ectopic pregnancy, T Testosterone, E_2_ Estradiol. **c**, **d** Violin plots and boxplots showing the distribution of Shannon diversity in different categories of contraception and delivery mode. Comparisons were investigated by linear regression models adjusting for covariates (see “Methods”). For all boxplots, the central line, box and whiskers represent the median, interquartile range (IQR), and 1.5 times the IQR, respectively. *, **, ***: FDR < 0.05, 0.01, and 0.001. **e** Venn diagram showing the variables identified as significantly associated with inter-individual variations of the vaginal microbiome by ADONIS, ANOSIM, db-RDA, and MRPP analysis based on at least three of four beta-diversity metrics. **f** Significant variables associated with microbiome variation based on Bray–Curtis distance (FDR < 0.1 & Adonis R^2^ > 0.001). The bar plot indicates the explained variation of each variable. The inner image shows variance in microbiome composition explained by eight phenotype categories. BV bacterial vaginosis, FSH follicle stimulating hormone, Neu abs neutrophil absolute count, E_2_ estradiol, Residence participants’ city of residence, VVC Vulvovaginal candidiasis, BMI Body mass index, AMH Anti-mullerian hormone, Sexual freq Sexual frequency, HGB Hemoglobin, Basal LH Basal luteinizing hormone, Douching freq Vaginal douching frequency, CS history Cesarean section history

We investigated the relationships between 81 host and environmental variables and the alpha diversity (the biological diversity within a sample) of the vaginal microbiome, and identified 13 variables significantly correlated with at least three of four alpha-diversity measures (FDR < 0.05; Fig. 2a, supplementary Table 3 for Shannon, Simpson, Chao1, and observed richness indices). The rarefaction curve of ASV richness reached a plateau, indicating that most of the microbial diversity had been captured (supplementary Fig. 1b–e). BV showed the strongest positive correlation with the alpha diversity (Spearman’s correlation, FDR < 0.05; Fig. 2b, supplementary Table 3). The microbial diversity increased as the disease state changed from normal to BV (supplementary Fig. 1b, c). Personal characteristics including age and BMI were positively correlated with the alpha diversity (Fig. 1b and supplementary Fig. 1d, e). As for lifestyles, women with higher exercise or sexual frequency had significantly higher alpha diversity levels. Compared with women who used intrauterine devices (IUDs), women who used condoms or did not use any contraceptive methods had lower alpha diversity (Fig. 2c, supplementary Table 4). Interestingly, alpha diversity was positively correlated with the parity numbers (total number of births). Women who have had a previous cesarean section or vaginal delivery had higher alpha diversity than women with no pregnancy history (Fig. 2d, supplementary Table 4). We added the statistically significant variables identified above from each respective phenotype category in the linear regression models (representative variables included age, BMI, education level, sampling month, parity, exercise frequency, and aspartate aminotransferase (AST) level from each category, see “Methods”, supplementary Table 5). The impact of variables such as BV, menopausal status, age, parity, serum estradiol (E_2_), BMI, sampling month, exercise and sexual frequency remained significant (FDR < 0.05, Fig. 2b, supplementary Table 4).

Analysis based on the four distance metrics (Bray–Curtis dissimilarity, weighted UniFrac, unweighted UniFrac, and Jaccard distances, Fig. 2e, f, supplementary Fig. 2a, supplementary Table 6a for four methods: permutation multivariate analysis of variance (ADONIS), analysis-of-similarity (ANOSIM), multi-response permutation procedure (MRPP), and distance-based redundancy analysis (db-RDA)) indicated that 24 variables were significantly associated with the beta diversity (microbial community difference between samples) of the vaginal microbiome (FDR < 0.1 in at least three of four methods based on no less than three distance metrics, Adonis R^2^ > 0.001). Among them, BV showed the strongest association, explaining 6% of the overall microbiome variation (Fig. 2f, supplementary Table 6a). Follicle-stimulating hormone (FSH), neutrophil absolute count, E_2_, age, parity, menopausal status, exercise frequency, and delivery mode were strong explanatory variables as well. Social factors, such as education, income, and profession also contributed to vaginal microbiome variation. Intriguingly, environmental variables, including sampling month and outdoor temperature, as well as participants’ city of residence in social factors, affected vaginal microbiome composition significantly. We calculated the total proportion of microbial composition variance explained by each category of phenotypes. The result showed that, in addition to the strongest impact from vaginal dysbiosis itself, the largest contribution came from lifestyle, laboratory measures, and social factors (Fig. 2f, supplementary Table 6b).

### Identification of microbial taxa associated with variables

**Fig. 3:**
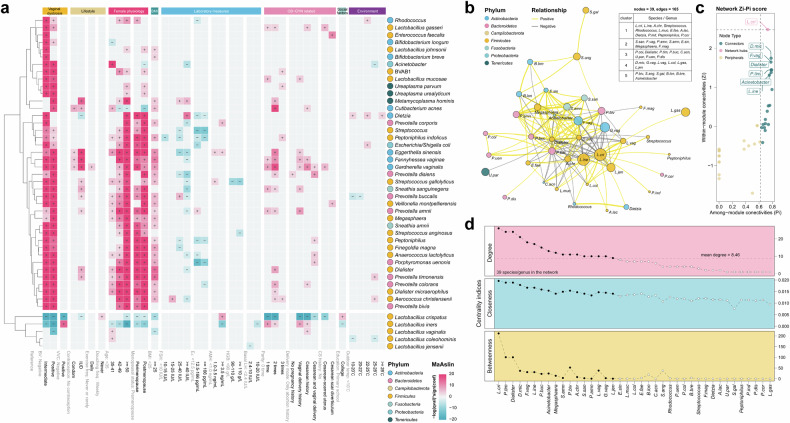
Identification of microbial taxa significantly associated with various variables. **a** Heatmap of the microbial taxa that were found to be significantly associated with different host or environmental variables using multivariate association with linear models (MaAsLin). Each level of every variable was compared with the reference level. Significant associations with FDR < 0.05 are marked with +/−. Blue and pink represent negative and positive correlations, respectively. BMI Body mass index, CS Cesarean section history, IUA Intrauterine adhesion, BV Bacterial vaginosis, VVC Vulvovaginal candidiasis, AMH Anti-mullerian hormone, E_2_ Estradiol, FSH Follicle stimulating hormone, Basal LH Basal luteinizing hormone, T Testosterone, HGB Hemoglobin. **b** SparCC network constructed by 45 identified microbial taxa. Microbial taxa are colored by phylum. The size of each node is proportional to relative abundance. Each edge represents a significant correlation with gray and yellow lines representing negative and positive correlations, respectively. The width of the edge indicates the strength of the sparCC correlation. The division of modules was calculated by the maximal greedy algorithm. **c** Zi-Pi plot showing the distribution of key species/genus based on their topological roles in the network. The threshold values of Zi and Pi for categorizing species/genus are 2.5 and 0.62, respectively. **d** Degree, closeness centrality, and betweenness centrality of each taxon in the network. The horizontal dashed line indicates the mean value for the degree of identified bacterial taxa

We next sought to identify the specific microbial taxa that contributed to the microbiome-variable associations. Microbiome multivariate association with linear models (MaAsLin) was applied to identify microbial species associated with the variables that showed significant association with beta diversity. After adjusting for the covariates (including age, BMI, residence, sampling month, delivery mode, sexual frequency, vulvovaginal candidiasis (VVC), and hemoglobin level, see “Methods”; supplementary Fig. 2b, supplementary Table 5), 45 microbial taxa were identified as being associated with at least one variable (FDR < 0.05, Fig. 3a, supplementary Table 7).

BV in the category of vaginal dysbiosis displayed the highest number of associations. Compared with the normal group, the abundances of *L. crispatus* and *L. iners* were lower in the BV group, while *G. vaginalis* and all other anaerobic and facultative bacteria were enriched (Fig. 3a). *L. crispatus* was also markedly lower in individuals affected by VVC. In contrast, a higher abundance of *L. iners* in women with VVC was observed.

Age and menopausal status were also strongly associated with the vaginal microbiota in a similar pattern (Fig. 3a). The abundances of core vaginal microbes *L. crispatus* and *L. iners* were lower, while those of *G. vaginalis* and *U. parvum* were significantly higher in women over 50 years old and in post-menopause women (FDR < 0.05, Fig. 3a, supplementary Table 7). The potential pathogenic genera *Fannyhessea* (formerly *Atopobium*), *Prevotella*, *Porphyromonas*, *Anaerococcus, Finegoldia*, *Dialister*, and *Streptococcus*^3^ were significantly higher in menopausal women and in women over 50 years old. Consistent with the age-related observations, a higher abundance of *L. crispatus* was identified in women with higher anti-müllerian hormone (AMH) levels, which is secreted by small, growing follicle,^22^ and is a known indicative marker for good ovarian reserve,^23,24^ while *L. iners* was negatively associated with AMH levels (Fig. 3a).

In particular, the core species *L. crispatus* was associated with twelve variables. Women with BMI over 25, frequent vaginal douching, IUD use, lower education level, multipara, and history of vaginal and/or cesarean delivery, had lower abundances of *L. crispatus*, while higher abundances of various bacteria that were reported as potential pathogenic, such as species from *Prevotella, Gardnerella* and *Atopobium* (*Fannyhessea*),^3,25^ were observed (FDR < 0.05, Fig. 3a).

We next conducted a network analysis comprising these 45 microbial taxa (Fig. 3b). The results showed that *L. crispatus*, *Prevotella timonensis*, *Dialister micraerophilus*, and *Fannyhessea vaginae* (formerly known as *Atopobium vaginae*^26^) ranked highest in the network centrality indices including degree (the number of connections to the node), closeness (the node’s distance to any other node), and betweenness (the number of shortest paths between any two nodes in the network passing through that node) (Fig. 3d, supplementary Table 8). *L. crispatus* emerged as the network hub (Fig. 3c), connecting extensively with other microbes both within its module and across different modules. This indicated its important biological impacts on community structure in the vaginal ecosystem.^27^

### Dynamic succession of the vaginal microbiome across the reproductive lifespan and menopause

**Fig. 4:**
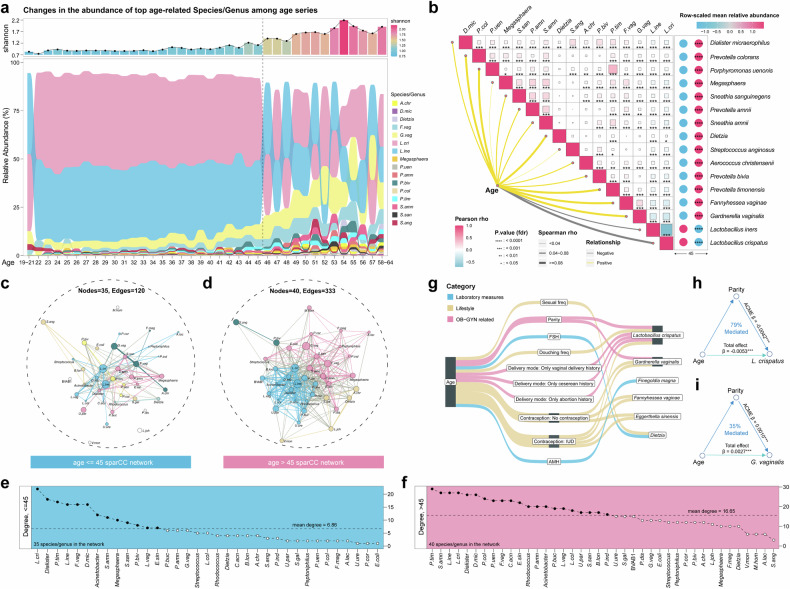
Dynamic variation of the vaginal microbiome across the different age groups. **a** Stack bar plot of the variation of average relative abundance of age-associated bacterial taxa across different age groups. The top 16 age-associated taxa determined by regression coefficient in MaAsLin are shown. The plot at the top denotes the average Shannon diversity of the age groups. **b** Relationships between age and the age-associated bacterial taxa. The Spearman correlations between the relative abundance of the top 16 age-associated taxa and age are shown as lines in the lower left region. The line width indicates the absolute value for the Spearman rho value. The line color indicates the direction of correlation (yellow for positive, gray for negative). The Pearson correlation between bacterial taxa is shown in the left plot. The size of the square in each cell is proportioned to the Pearson correlation r value. The dot plot at right indicates the mean relative abundance of taxa in women of different age groups (>45 vs. <=45). *, **, ***, **** indicate *t*-test *P* value < 0.05, 0.01, 0.001, 0.0001, respectively. **c**, **d** Microbiome networks of women in different age groups computed by sparCC. Analysis was conducted among 40 age-associated taxa determined by MaAslin. Nodes are colored according to the modules obtained from the maximal greedy algorithm within each network. The size of each node is proportional to the relative abundance of the taxa. Intra-module edges are colored by modules and inter-module edges are colored gray. The width of the lines reflects the strength of the correlations. **e**, **f** Degree of bacterial taxa of the microbiome network in different age groups. The taxa are ranked by the number of connections (i.e., degree). The horizontal dashed lines indicate the mean degree for bacterial taxa of the network in different age groups. **g**–**i** Sankey diagram showing the causal relationship of age-host variables-bacteria inferred by mediation analysis (ACME FDR < 0.05 & Total Effect FDR < 0.05 & Sensitivity Analysis rho > 0). Age-associated bacterial taxa and age-associated host variables were included in the mediation analysis. The beta coefficient and significance are labeled at the edge and the proportions of the mediation effect are labeled at the center. ***, FDR < 0.001

As age is robustly associated with the diversity and composition of the vaginal microbiome (Fig. 2b, f), we explored the chronological succession pattern of the top age-associated taxa (obtained by regression coefficients in MaAsLin) (Fig. 4a, b). The alteration trajectories of the vaginal microbiome with increasing age showed that *L. crispatus* and *L. iners* accounted for the largest portion of the vaginal microbiome and significantly decreased in women older than 45 years old, while the abundances of potentially pathogenic species such as *G. vaginalis*, *F. vaginae*, *Prevotella species*, and *Dialister micraerophilus*^3^ were significantly higher in women over 45 years old (Fig. 4a, b and supplementary Fig. 3a, d). Interestingly, there was a turning point at age 45 (Fig. 4a, supplementary Fig. 3a), after which bacterial composition began to exhibit undulating changes. *L. crispatus* and *L. iners* remained intermittently predominant and the abundances of other age-associated species also started to be higher in women around the age of 45 (Fig. 4a, b and supplementary Fig. 3a).

To determine the sequential changes in the microbial interaction across different age groups, we used SparCC^28^ to generate microbial correlation networks for women younger and older than 45, respectively (Fig. 4c, d, supplementary Fig. 3c, supplementary Table 9). The bacterial interplay in the women older than 45 (333 edges with 40 nodes) was much more complex than that in younger women (120 edges with 35 nodes), which indicated a more complicated, denser microbial network coupled with altered vaginal bacterial abundance when aging. Topologically, *L. crispatus* served as a hub species in women younger than 45, while there was no hub in women older than 45 (supplementary Fig. 4a). We next explored the modules, the highly interconnected sub-structures that represent ecological units within networks,^29^ in two age groups. Two main modules determined by the Greedy modularity algorithm^30^ were identified. The first module included the positive correlation between two potential pathogenic microbes: *G. vaginalis* and *F. vaginae*. The second module contained *L. crispatus* and *L. iners* (Fig. 4c, d and supplementary Fig. 3c). In women younger than 45, taxonomically related microbes tended to cluster in the same module and correlations were mostly positive, yet *L. crispatus* and *L. iners* were negatively connected. In women older than 45, a module contained taxa with more diverse biological classification and there were more competitive (negative) relationships (supplementary Fig. 3c), while *L. crispatus* and *L. iners* cooperated (positively correlated) with each other. In both age groups, *L. crispatus* and *L. iners* exerted strong antagonism against the potentially pathogenic *G. vaginalis* and *F. vaginae* module.^3,25^

The mean number of microbiome-microbiome interactions in the network was also higher in older women than in younger women (Fig. 4e, f, 16.65 vs. 6.86). Notably, within the vaginal flora of young women, the degree of *L. crispatus* was the highest, followed by *Dalister* and *P. timonensis*, which are associated with dysbiosis, while *L. iners* ranked after them. In contrast, in women over 45 years of age, along with an increase in abundance (Fig. 4b), the degree of BV-associated bacteria *P*. *timonensis* and *Sneathia amnii* was at the top of the community, sequentially followed by *L. iners* and *L. crispatus*.

The analysis of the predicted KEGG Orthology (KO) groups from the 16S rRNA gene showed marked differences in pathway distribution among different ages (supplementary Fig. 5b, c) and the abundances of many pathways turned at 45 years old (supplementary Fig. 5a, supplementary Table 10). The abundance change patterns of predicted KEGG pathways were determined. There were 63 pathways increased and 48 pathways decreased in women older than 45. For instance, the pathways associated with carbohydrate metabolisms such as glycosis/gluconeogenesis, pyruvate metabolism, glycerophospholipids metabolism^31^ and peptidoglycan biosynthesis, which have been linked to the probiotic effects of *Lactobacillus*,^19^ were enriched in women younger than 45, while the abundances of pathways involved in beta-Lactam resistance, proteasome,^32^ lipopolysaccharide biosynthesis, and bacterial invasion of epithelial cells, which are linked to bacterial survival and virulence, were enriched in women over 45.

We next explored what drove the age-related vaginal microbiome alteration. Cramer’s V correlation analysis among variables indicated age was most correlated with hormonal parameters including AMH, FSH, and E_2_ (FDR < 0.05, supplementary Fig. 2b, supplementary Table 5), each of which changed progressively after the age of 45 (supplementary Fig. 3b). This suggested that hormonal fluctuations may not be fully responsible for the observed microbial composition alteration with aging.

Then, we conducted mediation analyses to infer causal in silico relationships between age, vaginal microbiota, and host variables. We hypothesized that the impact of age on vaginal microbiota could be mediated by host variables. In total, 16 putative causal relationships were identified (ACME FDR < 0.05 and Total Effect FDR < 0.05, rho value > 0, Fig. 4g, supplementary Table 11). Among them, *L. crispatus* was involved with the highest number of relationships. The parity number exerted the strongest regulation of the aging effect on *L. crispatus* abundance (Fig. 4h, supplementary Table 11). The higher parity number contributed to lower *L. crispatus* abundance and higher *G. vaginalis* resulting from increasing age (Fig. 4h, i, supplementary Table 11). Additionally, delivery mode, lifestyles such as contraception method and douching frequency also mediated the impact of aging on the *L. crispatus* abundance (supplementary Table 11).

### Identifying vaginal Vagtiypes in the Chinese population and validation in different populations

**Fig. 5:**
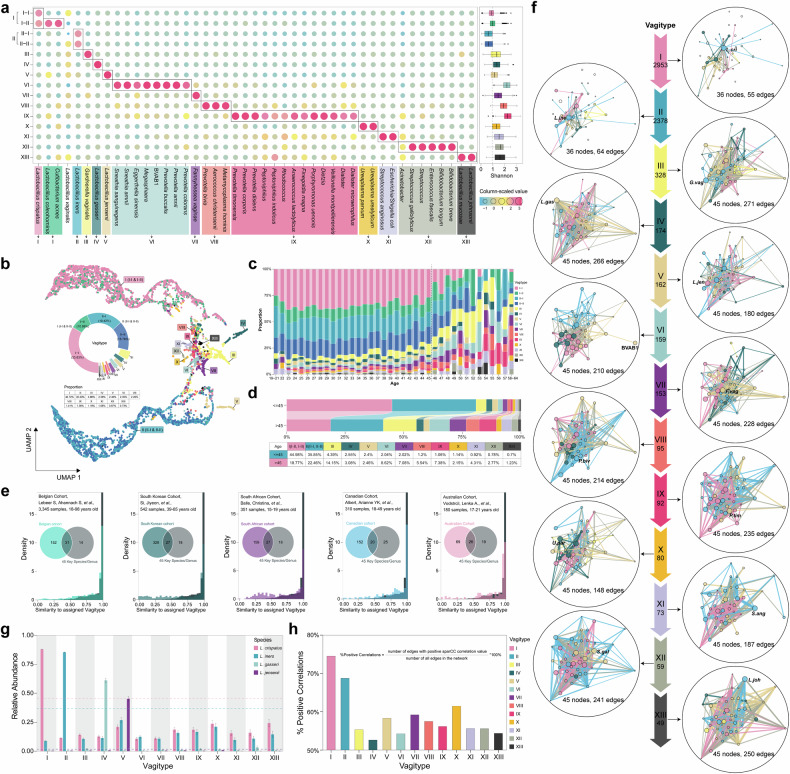
The Vagitypes in the VaMHP cohort. **a** Assignment of vaginal Vagitypes based on 45 host or environment-associated microbial taxa. A total of 15 groups, 13 vaginal Vagitypes were identified. Marker taxa enriched in the specific Vagitype (determined by NMF) were in the same color. The boxplots indicate Shannon diversity in each Vagitype. The central line, box and whiskers of boxplots represent the median, interquartile range (IQR), and 1.5 times the IQR, respectively. **b** UMAP (Uniform Manifold Approximation and Projection) plot presenting the distinct clustering of the Vagitypes in the VaMHP cohort and the proportion of each Vagitype. **c**, **d** Distribution of Vagitypes in women of different ages and age groups (<=45 vs. >45). **e** Validation of the Vagitypes defined from the VaMHP cohort in five independent populations. The similarity of each sample to its assigned Vagitype centroid was computed using Yue and Clayton’s θ index, a similarity measure based on relative abundances of shared and non-shared species (θ = 0: complete dissimilarity, θ = 1: identical communities). The similarity of each sample to its assigned Vagitype centroid in five validation cohorts is plotted as colored, normalized histograms and is compared to that of the VaMHP cohort (gray histogram). The Venn plot showed the shared and exclusive taxa between the taxa identified in the validated cohort and 45 taxa identified in the VaMHP cohort. **f** Microbial interactions of the 13 Vagitypes. The network was constructed by 45 identified host or environment-associated key microbial taxa. Microbial taxa are colored by modules obtained from the maximal greedy algorithm within each network. The size of each node is proportional to its relative abundance. Intra-module edges are colored by modules and inter-module edges are colored gray. The width of the lines reflects the strength of the sparCC correlations. **g** The relative abundance of four main *Lactobacillus* species, *L. crispatus*, *L. iners, L. gasseri*, and *L. jensenii* across the 13 Vagitypes. The horizontal dashed lines indicated the mean relative abundance of *L. crispatus* and *L. iners*. **h** The percentage of the positive correlations of the network in each Vagitype

Population stratification by microbiome classification can facilitate a better understanding of complex biological factors in health,^33^ and this has been an area of intense investigation in the vaginal microbiome.^2,13^ We next investigated the stratification of the vaginal microbiome in our cohort. The basis for community classification was defined as 45 variable-associated taxa identified by MaAsLin. Non-negative Matrix Factorization (NMF)^34^ was applied to 6755 vaginal microbiota profiles (supplementary Fig. 6), this identified 13 vaginal community types (henceforth termed “Vagitype”; Fig. 5a, supplementary Figs. 6, 7). UMAP (Uniform Manifold Approximation and Projection) plots show distinct clustering of the 13 Vagitypes (Fig. 5b).

Of the 13 Vagitypes, four harbored high abundances of *Lactobacillus*, dominated by *L. crispatus* in Vagitype I (subtype I-I with *Lactobacillus vaginalis*; subtype I-II with *Lactobacillus coleohominis* and *Cutibacterium acnes*), *L. iners* in II (subtype II-II with *Lactobacillus vaginalis*), *L*. *gasseri* in IV, *L. jensenii* in V (Fig. 5a, g). The abundances of core taxa *G. vaginalis* and *U. parvum* were enriched in Vagitype III and X, respectively. *F. vaginae* was enriched in Vagitype VII. The remaining Vagitypes were characterized by a wide range of other facultative and obligate anaerobic species. In addition to *F. vaginae*, other bacterial vaginalis associated bacteria including *Prevotella* spp*., Sneathia*, BVAB1, *Megasphera*, *Dialister*, and *Finegoldia* were enriched in Vagitype VI, VIII and IX (Fig. 5a, supplementary Table 12). Furthermore, aerobic vaginitis (AV) associated bacteria such as *Escherichia*/*Shigella coli, Enterococcus faecalis,* and *Streptococcus anginosus* were dominant bacteria in Vagitype XI and XII (Fig. 5a, supplementary Table 12). The distribution of Vagitypes was uneven, with *L. crispatus-* and *L. iners*-dominated Vagitypes I and II accounting for the largest portion (43.7% and 35.2%, respectively, Fig. 5b, supplementary Table 12).

We compared the potential functions of different Vagitypes (supplementary Fig. 8a), the 13 Vagitypes were divided into four clusters based on the abundance pattern of potential functional pathways. Vaginal types (I, II, IV, V) dominated by four common *Lactobacillus* species shared a similar metabolic pattern, such as more enriched pathways of galactose metabolism, glycolysis/gluconeogenesis, pyruvate metabolism, and pentose phosphate. Vagitypes III, VI, VII, and VIII, dominated by BV-associated bacteria, had similar metabolic patterns. The AV-associated bacteria dominated Vagitype XI and XII, although not in the same metabolic cluster, had enriched pathways of bacterial invasion of epithelial cells, beta-Lactam resistance, vibrio cholerae pathogenic cycle, and proteasome.^32^

We next explored the aging-related vaginal microbial alterations in the different Vagitypes. In line with previous observations of a depleted abundance of both *L. crispatus* and *L*. iners in women over 45, the proportion of Vagitype I (*L. crispatus*-dominant) and II (*L. iners*-dominant) decreased while the other eleven Vagitypes consistently showed an increasing trend (Fig. 5c). Of note, non-*Lactobacillus*-dominant Vagitypes were also enriched in older women (Fig. 5d), which may in part explain the high risk of ecological disturbance in elder women.

Network analysis showed microbial correlations were distinct among the 13 Vagitypes (Fig. 5f, supplementary Fig. 9). Compared to other Vagitypes, Vagitype I and II tended to have less complicated microbial interactions with a much lower degree (mean degree = 3.06 and 3.56 for Vagitype I and II, respectively; supplementary Fig. 9b). The networks of Vagitype I and II exhibited the highest proportions of positive inter-microbial correlations (74.5% and 68.8% for Vagitype I and II, respectively; Fig. 5h, supplementary Fig. 9a), which indicated a less stable community according to the ecological modeling that competitive interactions should promote stability of microbial communities.^35,36^ Based on the within module connectivity (Zi) and among module connectivity (Pi) of individual nodes in each network of thirteen Vagitypes, we then detected the module hubs (highly connect to microbes in their own modules), connectors (connect to other microbes in other modules), network hubs (act as both module hubs and connectors), and peripherals (only connect few microbes within modules). In *Lactobacillus*-dominant Vagitype I, II, and V, taxa such as *L. crispatus L. iners*, and *P. timonensis* served as network hubs (supplementary Fig. 4b). Meanwhile, few network hubs were found in other Vagitypes, with more connectors and peripheral taxa emerging instead (supplementary Fig. 4b). From an ecological perspective, peripherals can be eliminated without affecting the functions of the ecological structure, while the elimination of hubs would lead to the collapse of the entire network.^37^ Collectively, our network analysis indicated a more connected and stable microbial community in the non-*Lactobacillus* dominant Vagitypes. This suggested that the predominance of *Lactobacillus* spp. may render the vaginal ecosystem highly dependent and unstable.

Given that Vagitype I is the most prevalent Vagitype among the participants, and that previous reports suggest an *L. crispatus*-dominated community is representative of a healthy vaginal microbiome state,^19^ we adopted a multinomial logistic model to assess the phenotypic differences between Vagitype I and other Vagitypes (supplementary Fig. 10, see “Methods”). We found that women who had never delivered were significantly more prevalent in Vagitype I than in Vagitype II, III, IV, V, VII, X, and XIII. Women with lower douche frequencies were overrepresented in Vagitype I compared to Vagitype II and VI. Compared with Vagitype I, women with Vagitype III and VII adopted IUD more often. Additionally, women in Vagitype I were slightly younger than those assigned to Vagitype VI, VIII, and IX. Significant differences were also observed among different Vagitypes in income, education level, and sexual frequency (supplementary Fig. 10).

To explore whether the Vagitypes defined based on our data could be replicated in different populations, we validated the Vagitypes using 16S rRNA gene and cpn60 gene sequencing data from five different countries including Belgium (*n* = 3345),^14^ Australia (*n* = 180),^38^ Canada (*n* = 310),^39^ South Africa (*n* = 351),^40^ and South Korea (*n* = 542)^41^ (supplementary Table 13, Fig. 5e, supplementary Fig. 11, see “Methods”). Specifically, based on the VaMHP cohort, the reference abundance of previously identified 45 taxa for each Vagitype was constructed, namely reference centroid Vagitypes. Based on the reference, the Vagitype assignment was performed in the five validation cohorts. The results show that a considerable number of taxa in the external datasets overlapped with the 45 key vaginal microbiome taxa in the Chinese cohort (supplementary Fig. 11a–e). The distribution of similarity scores^13^ (see “Methods”) between the samples from each country and the matched 13 reference centroid Vagitypes showed that the South Korean, European, Canadian, and Australian datasets had high similarity with the VaMHP cohort (Fig. 5e). Albeit presenting different prevalence in regard to each Vagitype, a high proportion of participants (ranging from 54.5–77.5%) were dominated by *Lactobacillus* in these cohorts (supplementary Fig. 11f, g, i, j). The South African cohort also presented mainly *Lactobacillus*-dominant communities, but it showed a higher proportion with lower similarity to the VaMHP cohort and a higher prevalence of BV-associated bacteria dominated Vagitypes (supplementary Fig. 11h). These Vagitypes included *G. vaginalis* (Vagitype III), *P. amnii* (VI), *F. vaginae* (VII), *P. bivia* (VIII), *P. timonensis* (IX), which collectively accounted for 44.16%. This was consistent with the previous study indicating that the vaginal microbiome composition varied by ethnicity, with women of African ancestry being more likely to exhibit diverse bacterial communities.^2^

### Vagitype is associated with BV and the reproductive outcomes of IVF treatment

**Fig. 6:**
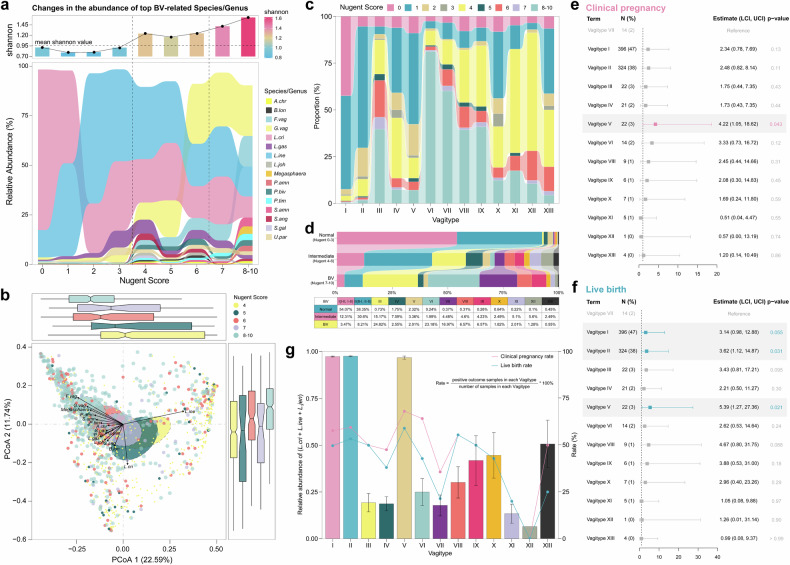
The relationship between BV, pregnancy outcome and vaginal microbiome variation. **a** Variation of average relative abundance of BV-associated bacterial taxa across different Nugent score groups. The top 16 BV-associated taxa determined by regression coefficient (absolute value) from MaAsLin are shown. The plot at the top denotes the average Shannon diversity across different Nugent score groups. **b** PCoA plot of samples with Nugent score 4–10 based on Bray–Curtis distance of microbial composition. The boxplots of PCo1 and PCo2 values of different Nugent groups are shown in the right and upper region. The central line, box and whiskers of boxplots represent the median, interquartile range (IQR), and 1.5 times the IQR, respectively. **c** Nugent score group distributions in different Vagitype. **d** Vagitype distributions in different BV groups. **e**, **f** Multivariate analysis for the IVF/ICSI outcome of live birth and clinical pregnancy in different Vagitypes. *P* values were calculated under a logistic regression model with Firth’s bias reduction method and adjusted for age, BMI, and the number of embryos transferred. OR odds ratio, CI confidence interval. **g** The total relative abundances of *L. crispatus*, *L. iners*, *L. jensenii*, and the live birth rate and clinical pregnancy rate across Vagitypes

To investigate potential associations between Vagitype and vaginal health, we first focused on the relationship between Vagitype and the common vaginal dysbiosis-BV. As the gold standard for BV diagnosis, the Nugent score assesses the proportion of *Lactobacillus* relative to *Gardnerella*, *Bacteroides*, and *Mobiluncus* based on Gram-stained vaginal smears, with categories including normal (0–3), intermediate (4–6), and BV (7–10).^42^ Alpha diversity, indicated by the Shannon index, increased with the Nugent score, particularly above Nugent 4 (Fig. 6a). Based on the alteration trend analysis of bacterial abundance along the increasing Nugent score, 43 BV-associated bacteria were clustered into four groups (supplementary Fig. 12): group 1 (*n* = 14, increased in BV positive women), group 2 (*n* = 12, increased in intermediate women), group 3 (*n* = 11, increased in both intermediate and BV positive women), and group 4 (*n* = 6, decreased in both intermediate and BV positive women). *L. crispatus* abundance peaked at Nugent 0 and declined as the score increased, while *L. iners* abundance peaked at Nugent 2^42^ (Fig. 6a, supplementary Fig. 12). *G. vaginalis* became the most abundant species from Nugent 6 (Fig. 6a), and there were no significant differences in the microbial composition between Nugent 6 (intermediate) and Nugent 7 (BV) (Fig. 6b, adonis *P* > 0.05), which may partly reflect the morphological similarity between *L. iners* and *G. vaginalis*.^43^ Accordingly, a significant portion (41.5%) of *G. vaginalis* dominat Vagitype III was assigned to the microscopical, ambiguous diagnosis of intermediate type (Fig. 6c). The proportion of BV was significantly different among the 13 Vagitypes (Fig. 6c, d). Normal samples (0–3 points) accounted for a large proportion of *Lactobacillus*-dominant Vagitypes I, II, IV, V, XIII, and *Ureaplasma*-dominant Vagitype X. BV samples (7–10 points) were more likely to be classified as Vagitype III, VI, VII, VIII, and IX, which were enriched with BV-associated bacteria (supplementary Table 12). AV-associated bacteria-dominated Vagitypes (XI, XII) were also more prone to be diagnosed as intermediate type.

To examine whether the Vagitypes at the molecular resolution provide a better prediction of reproductive outcomes than morphology-based BV diagnosis. We followed up a subset of 845 women from the current study cohort who underwent in vitro fertilization (IVF) treatment and fresh-embryo transfer after the baseline vaginal sampling (supplementary Table 14, see “Methods”). Overall, 486 (57.5%) of the 845 women had a clinical pregnancy, and the live birth rate was 50.1% (424/845). After adjusting for age, BMI, and the number of embryos transferred, no significant differences were observed between the normal group and the BV group in either clinical pregnancy rate or live birth rate (OR: 1.39, 1.65; 95% CI: 0.78–2.48, 0.92–3.01; supplementary Table 15). In contrast, *L. crispatus*-, *L. iners*-, and *L. jensenii*-abundant Vagitypes displayed high clinical pregnancy rates (Vagitype I: 57.83%, II: 59.57%, V: 68.18%, Fig. 6g) and live birth rates (Vagitype I: 49.75%, II: 53.40%, V: 59.09%, Fig. 6g), while *F. vaginae-*abundant Vagitype VII showed the lowest clinical pregnancy rate (35.71%) and live birth rate (21.43%, Fig. 6g). After adjusting for age, BMI, and the number of embryos transferred, the live birth rate was higher in Vagitype II and Vagitype V than in Vagitype VII (OR [95% CI]: 3.62 [1.12–14.87], 5.39 [1.27–27.36]; *P* = 0.031, *P* = 0.021; Fig. 6f, supplementary Table 15). In the adjusted model, *L. jensenii*-dominated Vagitype V also exhibited a higher clinical pregnancy rate than Vagitype VII (OR [95%CI]: 4.22 [1.05, 18.62]; *P* = 0.043, Fig. 6e, supplementary Table 15). Although we found an increasing trend for live birth rate in Vagitype I compared to Vagitype VII (49.75% vs 21.43%), differences between groups did not meet conventional levels of statistical significance (*P* = 0.055). However, we used the Boruta algorithm to identify significant microbial determinants in predicting live birth. The total abundance of *L. crispatus*, *L. iners*, and *L. jensenii* emerged as the most important microbial determinant (supplementary Fig. 13).

Functional level analysis showed that Vagitypes with better pregnancy outcomes (I, II, V) were enriched in pathways such as lactic acid-producing^44^ glycolysis/gluconeogenesis, pyruvate metabolism, and glycerophospholipid metabolism (supplementary Fig. 8a) and depleted of the pathway for lipopolysaccharide biosynthesis. The abundance of glycerophospholipids was found to be positively correlated with the abundance of vaginal *Lactobacillus*.^31^ Remarkably, the normal regulation of glycerophospholipids, whose accumulation was specifically observed in response to inoculation with *L. iners*, might be linked to successful embryo implantation.^31,45^ Collectively, these data indicate that Vagitypes denoting different vaginal microbiome structures play a vital role in the success of clinical pregnancy and live birth.

## Discussion

In line with previous reports,^14,46^ we observed that age and menopause status are strong influencers of the vaginal microbiome. Our results also agree with previous analyses of the association of parity, reproductive hormones, and BMI in the European population.^14,47,48^ In obese women who received bariatric surgery, those with the greatest weight loss 6-month post-surgery were most likely to have a *Lactobacillus*-dominant vaginal microbiota.^48^ Socioeconomic factors such as income/poverty level, education, and occupation were also demonstrated to play a role in shaping gut microbiome in large human populations.^49,50^ Our observation that social factors including education and income also had modest but significant associations with the β-diversity of vaginal microbiome corroborates previous studies that identified the impact of education^51,52^ and poverty level.^14,51^ Similar to our results of MaAsLin2 and diversity analysis, the associations between education level and overall microbiota variation and the abundance of *L. crispatus* were also observed in 50 asymptomatic Caucasian women.^52^ In a recent investigation of Belgian women, poverty level was positively linked to the vaginal Shannon diversity.^14^ Importantly, we discovered that the previously largely ignored factors of sampling month and participants’ residence had significant impacts on the vaginal microbiome. Likewise, it was reported that host location^49^ and sampling season^50^ exerted strong effects on human gut microbiome variations in large-scale population studies. The identified influence from geography and month may result in the differences of socioeconomic development, lifestyle and disease epidemiology at the regional and temporal level. These findings provided valuable clues about the impact of the spatiotemporal heterogeneity of the host on the vaginal bacterial communities. The impact variables we identified are pivotal in metadata collection and confounder-matching and for detecting the true differences between cases and controls in future microbiome studies. Based on our data, we adjusted for important covariates in our multivariate models and explored individual taxa associated with the variables. The identified associations between the health-promoting^18^ *L. crispatus*, potentially pathogenic species and host phenotypes provide the potential for intervening host variables to manipulate vaginal microbiome composition and modulate personalized female health.

With increasing age, women experience fluctuations in reproductive hormone levels. It has been previously hypothesized that the temporal variations of the vaginal microbiome during women’s reproductive stages and at menopause are mainly hormone-driven.^53^ We found that the turning point of microbiome composition occurred at age 45, which is earlier than the onset of rapid variation of reproductive hormone levels. This age is also much younger than the average age of natural menopause, which is commonly 49 years in Chinese women,^54^ suggesting a potential microbiome signature of female reproductive aging. This finding aligned with a smaller Chinese cohort, where notable fluctuations between 40 and 50 years in the vaginal microbiota were observed.^46^ Specifically, the positive correlation between age and microbial diversity was also similarly observed in this cohort.

In the microbiome network analysis, we detected two stably correlated modules comprising *L. crispatus* - *L. iners* and *G. vaginalis* - *F. vaginae* in women younger and older than 45, which echoed the findings of *L. crispatus* module and *Gardnerella* module in the European population.^14^ In both age groups, *L. crispatus* and *L. iners* were negatively correlated with potentially pathogenic *G. vaginalis* and *F. vaginae* modules. Notably, persistent microbial relationships that can distinguish multiple diseases were recently identified in the gut microbiome across different conditions and host environments,^55^ and it was hypothesized that stably correlated guild across diverse conditions are likely core members of health-relevant correlations.^55^ This suggests the stable interaction between *L. crispatus* and *G. vaginalis* may be an important indicator for the holistic vaginal health.

Previous studies have demonstrated that glycogen, as major carbon and energy source, fueled the growth of vaginal *Lactobacillus* spp.^56,57^ Higher glycogen levels are generally maintained across a woman’s reproductive stage and then decrease in menopause.^58^ The intermittently predominant abundance of *L. iners* in older women is in line with the notion that *L. iners* had remarkable adaptation to the changing carbohydrate sources in the aging vaginal niche.^59^ *L. iners* was reported to be unable to produce D-lactic acid and H_2_O_2_.^60,61^ A community dominated by *L. iners* is more prone to transition to an adverse microbiome state compared to a *L. crispatus*-dominated one.^62,63^ Hence it is considered that *L. iners* provided less overall protection to vaginal health. Compared to young women, the fewer interactions between probiotic *L. crispatus* and other taxa and increased interactions between *L. iners* and other BV-associated bacteria in menopausal women may contribute to their vulnerability to vaginal dysbiosis.

By leveraging our enriched time-matched metadata, we detected the complex, relationships between the vaginal microbiome and age. The mediation effect exerted by the parity on *L. crispatus* abundance we found was in line with the recent longitudinal study reporting that *L. crispatus* dominance was less observed over the post-partum year and past experiences of childbirth were associated with less *L. crispatus*-dominant vaginal bacterial communities.^64^ These results indicated that the process of female aging, which is not limited to hormonal changes, may be reflected in the vaginal microbial variation. However, the cross-sectional design of our study limited the ability to confirm causal relationships between age, host variables, and vaginal microbiome. The observed mediations in the current study should be interpreted with caution. Future large-scale population-based, longitudinal microbiome studies and more empirical research based on animal models are indispensable to elucidate the dynamic variation of vaginal microbiomes during aging and to establish causality.

Previous studies have shown that highly diversified vaginal microbiome types were associated with elevated pro-inflammatory cytokines.^65^ There were also differences in the risk of sexually transmitted diseases^66,67^ and preterm birth^8^ among different vaginal communities. To further investigate the relationship between health and the vaginal microbial community, we choose microbial species that were of great biological importance in the relationship with the host and adopted an unsupervised NMF classification method to stratify the vaginal community in the Chinese women. Various host profiles, which were commonly lacking in previous studies, were linked with the vaginal microbiome structure. The result showed that the Vagitype was dynamic and changed with the individual’s disease (BV) state, physiology, socioeconomic status, and lifestyle behaviors, which provide important clues to modify women’s vaginal microbiome. Classification based on microbial composition could potentially facilitate personalized management of one’s microbiome-related health. Interestingly, our network analysis among different vaginal bacterial communities indicated a more connected and stable microbial community in the non-*Lactobacillus* dominant Vagitypes as well as in women over 45. These results indicated that a stable community does not necessarily equate with ‘healthy’ in the vaginal microbiome.

In addition, five indicator vaginal taxa that contributed to community clustering corresponded with previously identified CSTs in North American women^2,13^: *L. crispatus*, *L. iners*, *L. jensenii*, *L. gasseri*, and *G. vaginalis*. Furthermore, our large dataset enables us to define Vagitypes which represent more uncommon vaginal microbial taxa (such as species from *Mobiluncus*, *Gemella*, *Ureaplasma*, and *Propionibacterium*) not noted by previous research.^2,13^ Validation in other racial groups showed our clustering strategy could be applied to most women from Caucasian, Asian, and African cohorts. This indicated that our data could serve as a valuable reference for future studies on vaginal microbiome classification.

We also compared the microscopic diagnosis for BV with 16S rRNA gene profiling of the vaginal microbiome. Our data showed Vagitype XI, and Vagitype XII, which were abundant of AV-associated Gram-negative aerobes, could not be classified properly in the Nugent system and were prone to be diagnosed as intermediate type. Our results indicated subgroup of intermediate samples structurally resembled the BV-positive group and there was a clear intrinsic relationship between BV and various Vagitypes. These results indicated that Vagitype classification resolves the vaginal microbiome community at a higher resolution than morphologically based Nugent score categories.

Moreover, we found that Vagitype was significantly associated with the live birth and clinical pregnancy rate. Prominent species in the poor-outcome Vagitype VII include the BV-associated *F. vaginae*, which has been linked to negative effects on reproductive health previously.^43,68^ Interestingly, *L. iners*- and *L. jensenii*-dominated Vagitypes II and V were most favorable for successful reproductive outcomes, while *L. crispatus*-dominated Vagitype only had an increasing trend for live birth compared to Vagitype VII. A recent study also demonstrated that women with *L. jensenii-*dominated community showed the highest pregnancy rate after IVF treatment.^69^ Of note, there were non-negligible abundances of *L. crispatus* and *L. iners* in the *L. jensenii*-dominated Vagitype at the same time. It has long been accepted that the dominance of *L. crispatus* in the vagina is linked to good health, while researchers showed *L. iners* played a controversial role in protecting vaginal health.^59^ The evidence of the positive effect of *L. iners* included that it was the only metronidazole-resistant-*Lactobacillus* species after BV treatment,^70^ and helped the recovery to a *Lactobacilli*-dominated microbial community.^71^ Considering the better adaptation to the depleted environmental carbohydrate sources of *L. iners*,^59^ and that an *L. crispatus*-dominated community was potentially variable and resilient,^62^ we speculated that a community state harboring moderate proportions of *L. crispatus* versus *L. iners* or other *Lactobacillus* species had better stress resistance than a community exhibiting extremely high abundance of *L. crispatus*. The cooperation among *Lactobacillus* species (especially *L. crispatus*, *L. iners*, and *L. jensenii*) may be the key to vaginal health and successful pregnancy outcomes. The beneficial effect of *Lactobacillus* spp. could be explained by mechanisms including lactic acid production that inhibits the growth of other potentially harmful bacteria,^72,73^ and its adhesion to the vaginal epithelial cells blocks the binding of pathogens.^74^ Additionally, it is reported that synthetic bacterial consortia constructed by *Lactobacillus* strains isolated from healthy women effectively modulate the immune response and restore vaginal microbiota in a mouse model of *G. vaginalis*-induced BV, but with a slightly weaker efficacy than vaginal microbiome transplantation (VMT).^75,76^ Increasing evidence from clinical trials has also demonstrated the efficacy of intravaginal probiotics including *L. crispatus* therapy in treating BV^77,78^ and VVC.^79^ Further understanding of microbial ecology, interbacterial interactions and host-bacterial interplay will help optimize the consortia compositions in which microbes can interact cooperatively and target pathogens more effectively. Albeit the underlying mechanism remains to be elucidated, our result suggested the potential of Vagitype assignment in personalized health management. Whether topical supplementation of specific *Lactobacillus* isolates from healthy women, synthetic bacterial consortia transplantation or VMT can restore vaginal equilibrium and promote improved pregnancy outcomes warrant further investigations in both mechanistic and clinical research.

This study has several limitations. First, we explored the vaginal microbiome by 16S rRNA gene sequencing. This methodology is limited to the study of bacterial composition and diversity, which does not cover other microorganisms such as fungi or protozoa. Future efforts evaluating a refined picture of microbiome composition and diversity should be obtained through shotgun metagenomics. Secondly, we chose 16S rRNA V1-V2 region sequencing as it has improved discrimination of the genus *Lactobacilli*.^80^ However, it has been reported that sequencing the V1-V2 region may underestimate the taxa abundance such as *G. vaginalis*, *B. bifidum,* and *Chlamydia trachomatis*.^81^ Caution is required when using 16S sub-regions sequencing to study microbial communities at the species level due to the potential to miss certain microbial diversity and decreased taxonomic accuracy. Lastly, it should be noted that some of the covariates that we did not identify, especially factors related to socioeconomic status such as access to healthcare, dietary habits, and hygiene practices, could be influencing the observed relationships between social factors and the microbiome. Additionally, despite the wide range of variables measured, the combination of all significant factors identified only explains around 13.96% of the total variability in microbiome composition, implying that the factors influencing the vaginal microbiome still need further investigation. As our findings are based on the Chinese population, the observed associations may not be extrapolatable to other ethnicities and warrant validation in other populations and ethnicities. Therefore, larger, longitudinal cohorts with more well-defined phenotypes and multi-omic data across different ethnicities are required to further elucidate meaningful host-microbiota links and promote the development of microbiome-targeting diagnosis and therapies.

In summary, our large-scale vaginal microbiome study revealed robust relationships between the vaginal microbiome and both host and environmental characteristics. Further mechanism studies will be needed to validate the discovered associations, and the Vagitype classification will likely facilitate the future development of microbiome-targeted clinical interventions.

## Materials and methods

### Study participants and metadata collection

Participants in this study were enrolled under Vaginal Microbiome Health Project (VaMHP). The participants were enrolled from the Center for Reproductive Medicine, Shandong University during the physical examination between March and September 2019, and between July and September 2020. The study procedures were approved by the Institutional Review Board of Reproductive Medicine, Shandong University (2019LSZ14). Written informed consent was acquired from all the participants.

Women with any of the following conditions were excluded: antibiotics usage within one month before swab sampling; vaginal douching or vaginal medication within one week; the presence of irritation around the genital area or abnormal vaginal discharge within one week; currently menstruating or vaginal bleeding; sexual intercourse within the current menstrual cycle. Within 10 months, 6786 samples were collected. We also collected 90 negative controls (including air swabs held in the examination room air for 60 seconds, blank swabs, and extraction buffer negative controls). The 16S rRNA sequencing was conducted on all samples, and a total of 6833 samples (comprising 6755 participant samples and 78 negative controls) were successfully sequenced and retained for subsequent analysis following rigorous quality control measures.

Participants were required to complete a detailed questionnaire to obtain participants basic demographic information, self-reported pregnancy history and menstruation conditions, general health, socioeconomic status and current life habits. Anthropometric measurements were done by trained staff. The obstetric and gynecological history as well as past medical history was obtained from the hospital information system. Participants’ clinical blood laboratory tests were taken at the lab department of the Center for Reproductive Medicine, Shandong University. Altogether, 81 time-matched host variables of eight categories including anthropometrics, social factors, lifestyle, environment, vaginal dysbiosis, female physiology, obstetrics and gynecology (OB-GYN)-related factors and laboratory measures were assessed in this study (Fig. 1a, supplementary Table 1).

### Vaginal swab processing

Vaginal swabs collection was performed by two gynecologists following a strict sampling protocol. The participant assumed a lithotomy position for a pelvic exam. Before any examination, the gynecologist inserted a sterile speculum into the vagina and used two sterile swabs to press against the posterior fornix for three seconds. After sampling, one of the swab heads was immediately cut off by a sterile surgical scissor and placed into a sterile tube. The tube was then placed on dry ice and transferred to a −80 °C freezer within 6 hours for subsequent 16S rRNA gene sequencing. The second swab was used for smear microscopy examinations conducted by a professional lab technician to evaluate Nugent score for BV diagnosis and Candida screening.

### DNA extraction and 16S rRNA amplicon sequencing

DNA extractions of all the swabs and 78 negative controls were performed using Magnetic Soil and Stool DNA Kits (TIANGEN BIOTECH, BEIJING) following the manufacturer’s instructions. DNA samples were stored at -80 °C. The V1-V2 region of the 16S rRNA gene was amplified using 27F and 338R primers (27F, 5’-AGAGTTTGATCCTGGCTCAG-3’, 338R, 5’-TGCTGCCTCCCGTAGGAGT-3’). This primer set has been commonly reported as informative for demonstrating vaginal bacterial taxon and diversity. The Illumina NovaSeq 6000 platform was employed for sequencing, resulting in a range of reads per sample from 48,673 to 96,989. The mean and median sequencing read depth were 78,301 and 79,047, respectively.

### 16S rRNA gene sequence data processing

The clean sequencing data (a total of 179 GB data with 535,028,707 reads) were handled by fastp software first and then processed using QIIME 2 software (version 2023.7.0).^82^ Reads with low quality (MaxEE value greater than 1.2) were filtered out, and the dada2 method^83^ was employed to obtain ASV-related data. The non-bacterial sequences were simultaneously eliminated during this procedure.

### ASV filtering and annotation

Following dada2 processing, a total of 42,634 bacterial ASVs and their representative sequences were initially obtained. Subsequently, ASVs with an absolute count sum less than 50 and prevalence below 1% were excluded, resulting in a remaining set of 452 ASVs. Thirdly, the R package decontam (version 1.22.0)^84^ was utilized for the identification and removal of contaminants, resulting in the acquisition of 310 ASVs. Finally, to address batch effects in the data, we employed the R package ConQuR (version 2.0)^85^ (supplementary Fig. 1f, g), which led to the identification of 274 ASVs for subsequent analysis.

The accuracy of species-level annotation for the representative sequences was improved by employing three distinct methods and databases: usearch 11 (RDP database version 18), QIIME 2 (SILVA database release 138), and STIRRUPS.^80^ The species annotation results were identified if they were consistent in at least two strategies; otherwise, the relevant sequence was annotated at the genus level. The representative sequences were aligned using the MAFFT software,^86^ and the resulting multiple sequence alignment was uploaded to the IQ-TREE^87^ website for phylogenetic tree construction.

### Vaginal microbiome diversity analysis

Then the alpha- and beta-diversity were calculated using the usearch 11 software. The ASV data were flattened to 10,000 by the *otutab_rare* function with parameter *sample_size* 10,000. A total of 143 samples were discarded in the diversity analysis after rarefication. The *alpha_div* function was utilized to compute the alpha diversity using default parameters, while the *alpha_div_rare* function was employed for calculating the rarefaction curves. Then the *calc_distmx* and *cluster_aggd* functions were used to construct a phylogenic tree using the representative sequences obtained from the QIIME 2. The *beta_div* function was utilized to compute the beta diversity using bray_curtis, jaccard, unifrac, and unifrac_binary metrics.

The Spearman’s rank correlation was employed to assess the association between alpha diversity (shannon_e value) and the continuous as well as ordinal variables. The *P* value was adjusted for multiple tests using the fdr method of False Discovery Rate (FDR) control.^88^ A statistically significant result was defined as an FDR value < 0.05. The correlations between the alpha-diversity and variables (significant variables of dysbiosis, socioeconomic, OB-GYN related factors, lifestyle and environment in Spearman’s rank correlation analysis and nominal variables including delivery mode, contraception, and profession) were further tested by fitting linear regression models, adjusting for covariates (supplementary Table 4). To assess the collinearity among the variables, pairwise Cramér’s V correlation was initially performed (supplementary Fig. 2, supplementary Table 5) to evaluate correlations between the variables. The statistically significant collinearity among variables was defined by Cramér’s V value > 0.3 and adjusted *P*-value < 0.05. In each phenotype category, the variable with a relatively high Spearman’s correlation coefficient with Shannon diversity and was not collinear with variables in other categories was selected as the representative variable of each category. Thus, the representative variables included age, BMI, education level, sampling month, parity, exercise frequency, and AST level. When analyzing the association between variable and alpha diversity, representative variables from other metadata categories were corrected in the linear regression models. An FDR value < 0.05 was considered statistically significant.

The associations between all 81 collected variables and the overall microbiota composition (beta-diversity analysis results based on Bray–Curtis, Jaccard, weighted UniFrac, and unweighted UniFrac metrics, respectively) were performed using the R package vegan with four methods: permutation multivariate analysis of variance (ADONIS), analysis-of-similarity (ANOSIM), multi-response permutation procedure (MRPP), and distance-based redundancy analysis (db-RDA). The *P* values in each method were obtained through 1000 permutations, and an initial statistical significance level of FDR < 0.1 was considered. Subsequently, the variables computed by each method were determined to be statistically significant based on at least three out of four metrics. The variables considered to be correlated with the vaginal microbiome were those supported in at least three methods and had an adonis R^2^ value greater than 0.001.

The total proportion of microbial composition variance explained by each category of phenotypes was calculated by multivariate ADONIS analyses which included all variables that showed significant association with overall microbiome composition in the univariate analyses from the respective category.

### MaAsLin analysis

In each phenotype category, the variable that was not collinear with variables in other categories and with relatively high ADONIS R^2^ was selected as the representative variable of each category. The representative variables included age, BMI, residence, sampling month, delivery mode, sexual frequency, VVC, and Hgb level.

To search for significant associations between microbes and explanatory variables, multivariable associations by linear models (MaAsLin) were performed using the MaAsLin2 (version 1.6.0)^89^ R package. When analyzing the association between each variable and the bacterial abundance, representative variables from other metadata categories were corrected in the MaAsLin model to eliminate the effect of confounding. An FDR < 0.05 was considered statistically significant.

### Network analysis of the vaginal microbiome

The SparCC method,^28^ implemented by fastSpar software^90^ with 1000 bootstraps, was utilized to establish correlation networks for the vaginal microbiome community and simultaneously estimate the corresponding *P* value in this analysis. The networks were constructed by including only significant correlations with *P* value < 0.01 and |cor| > 0.1. The modularity of the network was determined using the fast greedy clustering algorithm^30^ and visualized by the R package igraph (version 1.2.6).^91^ The centrality indices of nodes were computed and visualized using the R package qgraph (version 1.9.8).^92^

### Functional predictions of 16S rRNA datasets

The functional profiles of the microbial community were analyzed using the PICRUSt2 software (version 2.5.2)^93^ with default parameters based on the 274 ASVs and their corresponding representative sequences. The predicted KEGG Orthologs (KOs) obtained from PICRUSt2 were subsequently mapped to corresponding KEGG pathways to enhance the comprehensibility of the results. The samples were clustered using the umap method by the R package M3C (version 1.24.0), based on the log2 transformed functional value calculated by PICRUSt2. The relative abundance of predicted KEGG pathways was subjected to trend analysis among ordered groups using the R package ClusterGVis (version 0.1.1). The R package ‘Limma’ was used to test for differentially abundant KEGG pathways between women younger and women older than 45 years old.

### Mediation analysis

We conducted mediation analyses to infer in silico causal relationships between age, vaginal microbiota, and host variables using the R package “mediation” (version 4.5.0). Microbiome species which significantly associated with age in MaAsLin and host variables significantly associated with age were included in the analysis. The total effect, direct effect (ADE), and mediation effect (ACME) were estimated and the results were confirmed by the simulation exercises bootstrapped 1000 times. A candidate group was considered significant when ACME FDR < 0.05, Total Effect FDR < 0.05, and sensitivity rho > 0.

For continuous and ordinal variables, linear models were used in mediation analysis. Nominal variables were transferred to multiple dummy variables to fit binary logistic regression models in mediation analysis.

### Establishment of Vagitypes

The establishment of Vagitypes was derived from an unsupervised clustering analysis conducted on 6,755 samples. This process was conducted using non-negative matrix factorization by R package NMF (version 0.24.0),^94^ based on the relative abundance of 45 key vaginal species/genera obtained from MaAsLin2. The optimal solution was sought by setting a range of clusters from k = 2 to 20 in NMF. The results of the NMF were further examined to identify the optimal Vagitypes. The umap visualization coordinates were calculated using the R package M3C (version 1.24.0).

### Phenotypic comparisons of the Vagitype I and other Vagitypes

The multinomial logit model (nnet R package, *multinom* function, version 7.3) was used to assess the phenotypic differences of the variables between Vagitype I and other Vagitypes. The microbiome-associated variables (Age, BMI, douching frequency, education, sexual frequency, income, parity, contraception, sampling month) were included in the regression models. Forest plots were created with the ‘forestplot’ R package (version 2.0).

### Validation of the Vagitypes against other populations

To test whether the classifications of vaginal microbiome in the VaMHP cohort could be generalized to other populations, four independent 16S rRNA gene sequencing datasets and one cpn60 gene sequencing dataset of vaginal microbiome from public databases were retrieved (supplementary Table 13). The public datasets included: (1) 3345 samples from Belgian women (aged 18–98 years)^14^; (2) 180 samples from Australian university students with the ethnicity of Caucasian, Central and South-East Asian and Indian (aged 17–21 years)^38^; (3) 310 samples from non-pregnant, premenopausal Canadian women with diverse ethnicity distribution of the White, Asian, Black, Aboriginal and Hispanic population (aged 18–49 years)^39^; (4) 351 samples from South African adolescents receiving hormonal contraception (aged 15–19 years)^40^; and (5) 542 samples from twins and related and unrelated females in South Korea (mean age range from 39.76 to 65.06).^41^

The taxonomic assignment pipeline of the current study was performed for Belgian, South Korean, South African, and Australian datasets. The analysis of the Canadian dataset was based on the OTU table and taxonomic annotations provided in the published study. The validation process was referred to a recent study for vaginal microbial community classification.^13^ Briefly, the thirteen reference centroids were determined by the average relative abundances of the 45 key vaginal microbiome species/genus within each of the Vagitype in the VaMHP cohort. The samples from the outside datasets were assigned to Vagitypes according to the nearest centroid algorithm-based classification approach.^13^ A total of 6755 samples in the VaMHP cohort were reclassified as well. The similarity between the samples and each of the thirteen reference centroids is evaluated using Yue and Clayton’s θ index^95^ which considers the distance between communities being compared based on relative abundances of shared and non-shared species (θ = 0: complete dissimilarity, θ = 1: identical communities). Samples were assigned to the Vagitypes to which they had the highest similarity with the corresponding reference centroid.

### Reproductive outcome analysis

Data from 845 participants who underwent their first in vitro fertilization (IVF) or IVF with intracytoplasmic sperm injection (IVF-ICSI) and accepted fresh embryo transfer within 60 days from vaginal sampling were collected for analysis. The clinical pregnancy rate (the observation of a gestational sac on ultrasonography per embryo transfer cycle) and live birth rate were investigated. We performed multivariate logistic regression analysis with the reproductive outcome as the dependent variable and age, BMI, number of embryos transferred, and Vagitype/BV as covariates. We fitted the logistic regression model using Firth’s bias reduction method by the R package logistf (version 1.13), 95% CIs, and tests were computed by the profile penalized log-likelihood method. Forest plots were created with the R package forest (version 0.0.0.9000). To identify the significant microbial determinants in predicting live birth, we performed feature selection by the Boruta algorithm.^96^ The variables’ importance scores were calculated by the R Boruta (version 7.0.0) package.

## Supplementary information

Supplementary_Materials

supplementary table 1

supplementary table 2

supplementary table 3

supplementary table 4

supplementary table 5

supplementary table 6

supplementary table 7

supplementary table 8

supplementary table 9

supplementary table 10

supplementary table 11

supplementary table 12

supplementary table 13

supplementary table 14

supplementary table 15

## Data Availability

The clean microbiome sequencing data and limited metadata are available at the Genome Sequence Archive (https://ngdc.cncb.ac.cn/gsa-human/) under study accession ID: HRA002674.

## Code availability

Open-source codes and scripts used for the analyses or figures are available at the GitHub repository (https://github.com/jinansty/microbiology).
