# Supplementary material for: Population-level analyses identify host and environmental variables influencing the vaginal microbiome
Source: Signal Transduct Target Ther. 2025 Feb 19;10:64. doi: 10.1038/s41392-025-02152-8 (PMC11836416; doi:10.1038/s41392-025-02152-8)
Supplement: Supplementary file 13 — supplementary table 12 [file 41392_2025_2152_MOESM13_ESM.docx]

Table S12. Information for Vagitypes.

| **Vagitype (%)** | **Dominant species** | **Gram** | **Oxygen tolerance** | **Phylum** | **Health condition*** | **References** |
| --- | --- | --- | --- | --- | --- | --- |
| **I (43.72*%*)** | ***Lactobacillus crispatus*** | positive | anaerobe | Firmicutes | Dominated in the asymptomatic women or women without BV | ^1-3^ |
| I-I | *Lactobacillus crispatus* | **-** | **-** | **-** | **-** |  |
|  | *Lactobacillus vaginalis* | positive | anaerobe | Firmicutes | **-** |  |
| I-II | *Lactobacillus crispatus* | **-** | **-** | **-** | **-** |  |
|  | *Lactobacillus coleohominis* | positive | anaerobe | Firmicutes | Harbored in the healthy women | ^4,5^ |
|  | *Cutibacterium acnes* | positive | anaerobe | Actinobacteria | - |  |
| **II (35.20%)** | ***Lactobacillus iners*** | variable | anaerobe | Firmicutes | Dominated in the asymptomatic women or women without BV | ^1-3^ |
| II-I | *Lactobacillus iners* | **-** | **-** | **-** | **-** |  |
| II-II | *Lactobacillus iners* | **-** | **-** | **-** | **-** |  |
|  | *Lactobacillus vaginalis* | **-** | **-** | **-** | **-** |  |
| **III (4.86%)** | ***Gardnerella vaginalis*** | variable | anaerobe | Actinobacteria | BV-associated | ^2,6-8^ |
| **IV (2.58%)** | ***Lactobacillus gasseri*** | positive | anaerobic | Firmicutes | Dominated in the asymptomatic women | ^1,2^ |
| **V (2.40%)** | ***Lactobacillus jensenii*** | positive | anaerobic | Firmicutes | Dominated in the asymptomatic women | ^1-3^ |
| **VI (2.35%)** | ***Sneathia sanguinegens*** | negative | anaerobe | Fusobacteria | BV-associated | ^2,6,7^ |
|  | **BVAB1** | negative | uncultured | Firmicutes | BV-associated | ^2,6^ |
|  | ***Prevotella amnii*** | negative | anaerobe | Bacteroidetes | BV-associated | ^2,9^ |
|  | ***Prevotella buccalis*** | negative | anaerobe | Bacteroidetes | BV-associated | ^6,10^ |
|  | ***Prevotella colorans*** | negative | anaerobe | Bacteroidetes | - |  |
|  | ***Sneathia amnii*** | negative | anaerobe | Fusobacteria | BV-associated | ^11,12^ |
|  | ***Eggerthella sinensis*** | positive | anaerobe | Actinobacteria | BV-associated | ^2,6^ |
|  | ***Megasphaera*** | **-** | **-** | **-** | **-** |  |
| **VII (2.26%)** | ***Fannyhessea vaginae*** | positive | anaerobe | Actinobacteria | BV-associated | ^2,6-8^ |
| **VIII (1.41%)** | ***Prevotella bivia*** | negative | anaerobe | Bacteroidetes | BV-associated | ^8,13^ |
|  | ***Metamycoplasma hominis*** | NA | anaerobe | Tenericutes | BV-associated | ^14-18^ |
|  | ***Aerococcus christensenii*** | positive | microaerophilie | Firmicutes | - |  |
| **IX (1.36%)** | ***Prevotella timonensis*** | negative | anaerobe | Bacteroidetes | BV-associated | ^2,9^ |
|  | ***Prevotella corporis*** | negative | anaerobe | Bacteroidetes | BV-associated | ^13,19^ |
|  | ***Prevotella disiens*** | negative | anaerobe | Bacteroidetes | BV-associated | ^6,8,13^ |
|  | ***Dialister micraerophilus*** | negative | anaerobe | Firmicutes | BV-associated | ^2,7,9^ |
|  | ***Dialister*** | **-** | **-** | **-** | **-** |  |
|  | ***Peptoniphilus indolicus*** | positive | anaerobe | Firmicutes | BV-associated | ^20^ |
|  | ***Peptoniphilus*** | **-** | **-** | **-** | **-** |  |
|  | ***Rhodococcus*** | **-** | **-** | **-** | **-** |  |
|  | ***Anaerococcus lactolyticus*** | positive | anaerobe | Firmicutes | - |  |
|  | ***Finegoldia magna*** | positive | anaerobe | Firmicutes | - |  |
|  | ***Porphyromonas uenonis*** | negative | anaerobe | Bacteroidetes | - |  |
|  | ***Veillonella montpellierensis*** | negative | anaerobe | Firmicutes | - |  |
|  | ***Dietzia*** | **-** | **-** | **-** | **-** |  |
| **X (1.18%)** | ***Ureaplasma urealyticum*** | NA | anaerobe | Tenericutes | BV-associated | ^18,21-23^ |
|  | ***Ureaplasma parvum*** | NA | microaerophile | Tenericutes | - |  |
| **XI (1.08%)** | ***Escherichia/Shigella coli*** | negative | facultative anaerobe | Proteobacteria | AV-associated | ^24,25^ |
|  | ***Streptococcus anginosus*** | positive | facultative anaerobe | Firmicutes | AV-associated | ^26^ |
|  | ***Acinetobacter*** | **-** | **-** | **-** | **-** |  |
| **XII (0.87%)** | ***Enterococcus faecalis*** | positive | facultative anaerobe; microaerophile | Firmicutes | AV-associated | ^24,25^ |
|  | ***Bifidobacterium breve*** | positive | anaerobe | Actinobacteria | Harbored in the healthy women | ^3,9^ |
|  | ***Bifidobacterium longum*** | positive | anaerobe | Actinobacteria | Harbored both in the healthy women and BV women | ^27,28^ |
|  | ***Streptococcus gallolyticus*** | positive | anaerobe | Firmicutes | - |  |
|  | ***Streptococcus*** | **-** | **-** | **-** | **-** |  |
| **XIII (0.73%)** | ***Lactobacillus mucosae*** | positive | anaerobe | Firmicutes | - |  |
|  | ***Lactobacillus johnsonii*** | positive | anaerobe | Firmicutes | Harbored in the healthy women | ^29,30^ |

*Health condition was supported by more than two published references.

1 Ravel, J. *et al.* Vaginal microbiome of reproductive-age women. *Proc Natl Acad Sci U S A* **108 Suppl 1**, 4680-4687, doi:10.1073/pnas.1002611107 (2011).

2 Srinivasan, S. *et al.* Bacterial communities in women with bacterial vaginosis: high resolution phylogenetic analyses reveal relationships of microbiota to clinical criteria. *PLoS One* **7**, e37818, doi:10.1371/journal.pone.0037818 (2012).

3 Chaban, B. *et al.* Characterization of the vaginal microbiota of healthy Canadian women through the menstrual cycle. *Microbiome* **2**, 23, doi:10.1186/2049-2618-2-23 (2014).

4 Pramanick, R., Nathani, N., Warke, H., Mayadeo, N. & Aranha, C. Vaginal Dysbiotic Microbiome in Women With No Symptoms of Genital Infections. *Front Cell Infect Microbiol* **11**, 760459, doi:10.3389/fcimb.2021.760459 (2021).

5 Amin, M. E., Azab, M., Hanora, A., Atwa, K. & Shabayek, S. Compositional Changes in the Vaginal Bacterial Microbiome of Healthy Pregnant Women across the Three Gestational Trimesters in Ismailia, Egypt. *Microorganisms* **11**, doi:10.3390/microorganisms11010139 (2023).

6 Fredricks, D. N., Fiedler, T. L. & Marrazzo, J. M. Molecular identification of bacteria associated with bacterial vaginosis. *N Engl J Med* **353**, 1899-1911, doi:10.1056/NEJMoa043802 (2005).

7 Dols, J. A. *et al.* Molecular assessment of bacterial vaginosis by Lactobacillus abundance and species diversity. *BMC Infect Dis* **16**, 180, doi:10.1186/s12879-016-1513-3 (2016).

8 Dols, J. A. *et al.* Microarray-based identification of clinically relevant vaginal bacteria in relation to bacterial vaginosis. *Am J Obstet Gynecol* **204**, 305 e301-307, doi:10.1016/j.ajog.2010.11.012 (2011).

9 Xia, Q. *et al.* Identification of vaginal bacteria diversity and it's association with clinically diagnosed bacterial vaginosis by denaturing gradient gel electrophoresis and correspondence analysis. *Infect Genet Evol* **44**, 479-486, doi:10.1016/j.meegid.2016.08.001 (2016).

10 Srinivasan, S. & Fredricks, D. N. The human vaginal bacterial biota and bacterial vaginosis. *Interdisciplinary perspectives on infectious diseases* **2008**, 750479, doi:10.1155/2008/750479 (2008).

11 Carter, K. A. *et al.* Associations Between Vaginal Bacteria and Bacterial Vaginosis Signs and Symptoms: A Comparative Study of Kenyan and American Women. *Frontiers in Cellular and Infection Microbiology* **12**, doi:10.3389/fcimb.2022.801770 (2022).

12 Łaniewski, P. & Herbst-Kralovetz, M. M. Bacterial vaginosis and health-associated bacteria modulate the immunometabolic landscape in 3D model of human cervix. *npj Biofilms and Microbiomes* **7**, doi:10.1038/s41522-021-00259-8 (2021).

13 Hillier, S. L., Krohn, M. A., Rabe, L. K., Klebanoff, S. J. & Eschenbach, D. A. The normal vaginal flora, H2O2-producing lactobacilli, and bacterial vaginosis in pregnant women. *Clin Infect Dis* **16 Suppl 4**, S273-281, doi:10.1093/clinids/16.supplement_4.s273 (1993).

14 Challa, A. *et al.* Multi‐omics analysis identifies potential microbial and metabolite diagnostic biomarkers of bacterial vaginosis. *Journal of the European Academy of Dermatology and Venereology*, doi:10.1111/jdv.19805 (2024).

15 Cox, C., Watt, A. P., McKenna, J. P. & Coyle, P. V. Mycoplasma hominis and Gardnerella vaginalis display a significant synergistic relationship in bacterial vaginosis. *European Journal of Clinical Microbiology & Infectious Diseases* **35**, 481-487, doi:10.1007/s10096-015-2564-x (2016).

16 Bautista, C. T. *et al.* Bacterial vaginosis: a synthesis of the literature on etiology, prevalence, risk factors, and relationship with chlamydia and gonorrhea infections. *Military Medical Research* **3**, doi:10.1186/s40779-016-0074-5 (2016).

17 Plummer, E. L. *et al.* Are Mycoplasma hominis, Ureaplasma urealyticum and Ureaplasma parvum Associated With Specific Genital Symptoms and Clinical Signs in Nonpregnant Women? *Clinical Infectious Diseases* **73**, 659-668, doi:10.1093/cid/ciab061 (2021).

18 Hill, G. B. The microbiology of bacterial vaginosis. *American Journal of Obstetrics and Gynecology* **169**, 450-454, doi:10.1016/0002-9378(93)90339-k (1993).

19 Wertz, J., Isaacs-Cosgrove, N., Holzman, C. & Marsh, T. L. Temporal Shifts in Microbial Communities in Nonpregnant African-American Women with and without Bacterial Vaginosis. *Interdisciplinary perspectives on infectious diseases* **2008**, 181253, doi:10.1155/2008/181253 (2008).

20 Ji, C., Xu, F., Wang, Y. & Qin, Y. Peptoniphilus indolicus infection in a pregnant woman: a case report. *Curr Med Res Opin* **38**, 1439-1442, doi:10.1080/03007995.2022.2072091 (2022).

21 Ness, R. B. *et al.* A cluster analysis of bacterial vaginosis-associated microflora and pelvic inflammatory disease. *American Journal of Epidemiology* **162**, 585-590 (2005).

22 Abou Chacra, L., Fenollar, F. & Diop, K. Bacterial Vaginosis: What Do We Currently Know? *Frontiers In Cellular and Infection Microbiology* **11**, 672429, doi:10.3389/fcimb.2021.672429 (2021).

23 Hillier, S. L., Krohn, M. A., Rabe, L. K., Klebanoff, S. J. & Eschenbach, D. A. The normal vaginal flora, H2O2-producing lactobacilli, and bacterial vaginosis in pregnant women. *Clinical Infectious Diseases : an Official Publication of the Infectious Diseases Society of America* **16 Suppl 4**, S273-S281 (1993).

24 Donders, G. G. *et al.* Definition of a type of abnormal vaginal flora that is distinct from bacterial vaginosis: aerobic vaginitis. *BJOG : an international journal of obstetrics and gynaecology* **109**, 34-43, doi:10.1111/j.1471-0528.2002.00432.x (2002).

25 Donders, G. G. G., Bellen, G., Grinceviciene, S., Ruban, K. & Vieira-Baptista, P. Aerobic vaginitis: no longer a stranger. *Res Microbiol* **168**, 845-858, doi:10.1016/j.resmic.2017.04.004 (2017).

26 Tao, Z. *et al.* The Pathogenesis Of Streptococcus anginosus In Aerobic Vaginitis. *Infection and Drug Resistance* **12**, 3745-3754, doi:10.2147/IDR.S227883 (2019).

27 Freitas, A. C. & Hill, J. E. Quantification, isolation and characterization of Bifidobacterium from the vaginal microbiomes of reproductive aged women. *Anaerobe* **47**, 145-156, doi:10.1016/j.anaerobe.2017.05.012 (2017).

28 Utto, P., Teanpaisan, R., Piwat, S. & Chandeying, V. Assessment of Prevalence, Adhesion and Surface Charges of Bifidobacterium spp. Isolated from Thai Women with Bacterial Vaginosis and Healthy Women. *J Med Assoc Thai* **100**, 1-8 (2017).

29 Ahire, J. J. *et al.* In Vitro Assessment of Lactobacillus crispatus UBLCp01, Lactobacillus gasseri UBLG36, and Lactobacillus johnsonii UBLJ01 as a Potential Vaginal Probiotic Candidate. *Probiotics and Antimicrobial Proteins* **15**, 275-286, doi:10.1007/s12602-021-09838-9 (2023).

30 Zhang, R. *et al.* Qualitative and semiquantitative analysis of Lactobacillus species in the vaginas of healthy fertile and postmenopausal Chinese women. *Journal of Medical Microbiology* **61**, 729-739, doi:10.1099/jmm.0.038687-0 (2012).
