# Supplementary material for: Population-level analyses identify host and environmental variables influencing the vaginal microbiome
Source: Signal Transduct Target Ther. 2025 Feb 19;10:64. doi: 10.1038/s41392-025-02152-8 (PMC11836416; doi:10.1038/s41392-025-02152-8)
Supplement: Supplementary file 1 — Supplementary_Materials [file 41392_2025_2152_MOESM1_ESM.docx]

Supplementary Materials for

**Population-level analyses identify host and environmental variables influencing the vaginal microbiome**

Lang Qin,^1,2,3,4,5#^ Tianyong Sun,^6,7,8#^ Xiao Li,^1,2,3,4,5#^ Shigang Zhao,^1,2,3,4,5#*^ Zheng Liu,^1,2,3,4,5^ Changlong Zhang,^1,2,3,4,5^ Congcong Jin,^1,9^ Yanqi Xu,^1,10^ Xuan Gao,^1,2,3,4,5^ Yongzhi Cao,^1,2,3,4,5^ Jiaojiao Wang,^1,2,3,4,5^ Ting Han,^1,2,3,4,5^ Lei Yan,^1,2,3,4,5^ Jialun Song,^1,2,3,4,5^ Fangfang Zhang,^1,2,3,4,5^ Feifei Liu,^1,2,3,4,5^ Yousheng Zhang,^1,2,3,4,5^ Yuzhen Huang,^1,2,3,4,5^ Yuping Song,^1,2,3,4,5^ Yanjun Liu,^1,2,3,4,5^ Jing Zhang,^1,2,3,4,5^ Xiuqing Zhang,^1,2,3,4,5^ Zhina Yao,^1,2,3,4,5^ Honglei Chen,^1,2,3,4,5^ Zhenzhen Zhang,^1,2,3,4,5^ Shengrui Zhao,^1,2,3,4,5^ Yuhan Feng,^1,2,3,4,5^ Ya-nan Zhang,^1,2,3,4,5^ Qian Yu,^1,2,3,4,5^ Fang Cao,^1,2,3,4,5^ Lijuan Zhao,^1,2,3,4,5^ Lei Xie,^1,2,3,4,5^ Ling Geng,^1,3,11^* Qiang Feng,^7,8,9,12^* Han Zhao,^1,2,3,4,5^* Zi-Jiang Chen^1,2,3,4,5,13,14^*

Correspondence to: Shigang Zhao (zsg0108@126.com); Ling Geng (gengling78@126.com); Qiang Feng (fengqiang@sdu.edu.cn); Han Zhao (hanzh80@sdu.edu.cn);

Zi-Jiang Chen (chenzijiang@hotmail.com)

**This PDF file includes:**

Supplementary Figures 1 to 13

Other Supplementary Materials for this manuscript include the following:

Excel spreadsheet documents for Supplementary Tables 1 to 15

**Figure S1. Workflow of the sample collection, 16S rRNA amplicon sequencing, and bioinformatic analysis in the VaMHP cohort study.**

a, The analysis pipeline and the workflow of the study.

b-c, The rarefaction curve of vaginal microbiome in women with different BV diagnoses.

d-e, The rarefaction curve of vaginal microbiome in women with different age groups.

f-g, Evaluation of batch effects removal on the VaMHP data via ConQuR. PCoA plots cluster by batch ID based on Aitchison distance.

**Figure S2. Variables significantly associated with inter-individual microbiome variations in the VaMHP cohort.** Variables significantly associated with inter-individual variations of vaginal microbiome identified by ANOSIM, ADONIS, db-RDA, and MRPP analysis based on four beta-diversity metrics (FDR < 0.1).

a, A total of 24 variables associated with microbiome variation (based on four distances) determined by at least three out of four methods of beta diversity.

b, Correlations between variables significantly impacted microbiome variation. Heatmap shows the Cramer’s V correlation between variables significantly impacted microbiome variation. Correlation with FDR < 0.05 and Cramer’s V > 0.2 is considered significant.

**Figure S3. The abundance variation of vaginal microbes across different age groups.**

a, The abundance changing patterns of age-related vaginal microbes obtained by MaAsLin2 with the increase of age performed by the R package ClusterGVis.

b, Fluctuations of reproductive hormones across the female reproductive lifespan. Dots denote the average level of reproductive hormones of women in each age group. Error bars show standard error for the hormone levels of women in each group.

c, Discrepancy of microbiome ecological networks between women in different age groups. The nodes in the network are colored by phylum. The size of nodes represents the mean relative abundance of the taxa. The edge width reflects the strength of correlations. The edge color denotes the direction of correlations, with yellow and grey representing positive and negative correlations, respectively. The proportion of positive edges is shown in the pie chart.

d, Differential bacterial taxa between women younger and older than 45 tested by t-test. ****: p < 0.0001.

**Figure S4. The topological roles of the vaginal microbes in the ecological networks of women in different age Groups (a), and Vagitype groups (b).** Zi (within-module connectivity) and Pi (among-module connectivity) were calculated to categorize the nodes. The nodes with both high within-module and among-module connectivity were defined as network hubs (Zi > 2.5, Pi > 0.62). Nodes with high among-module connectivity but low within-module connectivity were defined as connectors (Zi < 2.5, Pi > 0.62). Nodes with low among-module connectivity but high within-module connectivity were defined as module hubs (Zi > 2.5, Pi < 0.62). The nodes with both low within-module and among-module connectivity were defined as peripherals (Zi < 2.5, Pi < 0.62).

**Figure S5. The dynamic succession of the predicted functions of the vaginal microbiome with increasing age.**

a, The changing patterns of vaginal predicted function with the increase of age identified by the R package ClusterGVis. Two distinct clusters of KEGG pathways and correspondingly two changing patterns were detected.

b-c, UMAP distribution of predicted functions of the vaginal microbiome among the 6,755 participants in the VaMHP cohort. The samples are colored by age and different age groups.

**Figure S6. The assignment of Vagitypes with identified host or environment-associated microbes (n = 45) using NMF.**

a, The best value for NMF factorization rank is surveyed within the range of 2 to 20 based on the cophenetic coefficient.

b, The marker microbes for each Vagitype determined by NMF.

c, The consensus matrix for the clusters of vagitypes. A total of 15 cluster groups were assigned. Basis 10 and 7 were assigned as Vagitype I-I and Vagitpe I-II, respectively, and collectively constituted Vagitype I. Basis 12 and 4 were assigned as Vagitype II-I and Vagitpe II-II, respectively and collectively constituted Vagitype II.

d, The taxonomic composition of samples in the 13 Vagitypes classified by NMF clustering in the VaMHP cohort.

**Figure S7. The taxonomic composition of 6,755 vaginal microbial communities based on the identified host or environment-associated microbes (n = 45).** Thirteen vaginal community types (Vagitypes) were identified. Each column represents an individual. Samples are grouped according to assigned Vagitype. The plot at the bottom indicated Shannon diversity corresponding to each column (sample).

**Figure S8. The predicted KEGG pathways of the vaginal microbiome with different Vagitypes.**

a, The changing patterns of the vaginal predicted function of different Vagitypes identified by the R package ClusterGVis.

b, UMAP distribution of predicted functions of the vaginal microbiome in women with different Vagitypes.

**Figure S9. Discrepancy of microbiome ecological networks among women with different Vagitypes.**

a, The network was constructed by 45 identified host or environment-associated microbial taxa for each Vagitype. Microbial taxa and adjacent edges are colored by modules calculated by the maximal greedy algorithm. The size of the nodes is proportional to the average relative abundance. The width of the edges indicates the strength of the sparCC correlations. The proportion of positive edges of each network is shown in the pie chart.

b, Degree of bacterial taxa in the microbiome networks of 13 Vagitype groups. The taxa are ranked by the number of connections (i.e., degree). The horizontal dashed lines indicate the mean degree for bacterial taxa of the network in different Vagitype groups.

**Figure S10. Forest plots for phenotypic comparisons between Vagitype I (*L. crispatus* dominant) and other Vagitypes.** The multinomial logit model was used and adjusted for microbiome-associated variables (see Methods).

**Figure S11. Validation of the Vagitypes defined from the VaMHP cohort in five independent populations.**

a-e, UMAP distribution of the Vagitypes of the vaginal microbiome in women from (a) Belgian cohort, (b) South Korean cohort, (c) South African cohort, (d) Canadian cohort, (e) Australia cohort.

f-j, The taxonomic composition of samples grouped by Vagitype assignment in (f) Belgian cohort, (g) South Korean cohort, (h) South African cohort, (i) Canadian cohort, (j) Australia cohort.

**Figure S12. Dynamic alteration of the vaginal microbiome along with the Nugent score.** The changing patterns of significant BV-related vaginal microbes (obtained from MaAsLin2) abundances with increasing Nugent score identified by the R package ClusterGVis.

**Figure S13. Variable importance scores from the Boruta algorithm.** Results from Boruta analysis modeling live birth as the dependent variable. Box plots show the estimated importance of total relative abundances of *L. crispatus*, *L. iners*, and *L. jensenii*; *L. crispatus* and *L. iners*; *L. iners* and *L. jensenii*; and the importance of abundances of *L. crispatus*, *L. iners*, and *L. jensenii* individually.
